# Outcomes After Initiation of Medications for Alcohol Use Disorder at Hospital Discharge

**DOI:** 10.1001/jamanetworkopen.2024.3387

**Published:** 2024-03-29

**Authors:** Eden Y. Bernstein, Travis P. Baggett, Shrunjal Trivedi, Shoshana J. Herzig, Timothy S. Anderson

**Affiliations:** 1 Division of General Internal Medicine, Massachusetts General Hospital, Boston,; 2 Harvard Medical School, Boston, Massachusetts; 3 Institute for Research, Quality, and Policy in Homeless Health Care, Boston Health Care for the Homeless Program, Boston, Massachusetts; 4 Division of General Medicine, Department of Medicine, Beth Israel Deaconess Medical Center, Boston, Massachusetts; 5 Division of General Internal Medicine, University of Pittsburgh, Pittsburgh, Pennsylvania

## Abstract

**Importance:**

US Food and Drug Administration–approved medications for alcohol use disorder (MAUD) are significantly underused. Hospitalizations may provide an unmet opportunity to initiate MAUD, but few studies have examined clinical outcomes of patients who initiate these medications at hospital discharge.

**Objective:**

To investigate the association between discharge MAUD initiation and 30-day posthospitalization outcomes.

**Design, Setting, and Participants:**

This cohort study was conducted among patients with Medicare Part D who had alcohol-related hospitalizations in 2016. Data were analyzed from October 2022 to December 2023.

**Exposures:**

Discharge MAUD initiation was defined as oral naltrexone, acamprosate, or disulfiram pharmacy fills within 2 days of discharge.

**Main outcomes:**

The primary outcome was a composite of all-cause mortality or return to hospital (emergency department visits and hospital readmissions) within 30 days of discharge. Secondary outcomes included these components separately, return to hospital for alcohol-related diagnoses, and primary care or mental health follow-up within 30 days of discharge. Propensity score 3:1 matching and modified Poisson regressions were used to compare outcomes between patients who received and did not receive discharge MAUD.

**Results:**

There were 6794 unique individuals representing 9834 alcohol-related hospitalizations (median [IQR] age, 54 [46-62] years; 3205 hospitalizations among females [32.6%]; 1754 hospitalizations among Black [17.8%], 712 hospitalizations among Hispanic [7.2%], and 7060 hospitalizations among White [71.8%] patients). Of these, 192 hospitalizations (2.0%) involved discharge MAUD initiation. After propensity matching, discharge MAUD initiation was associated with a 42% decreased incidence of the primary outcome (incident rate ratio, 0.58 [95% CI, 0.45 to 0.76]; absolute risk difference, −0.18 [95% CI, −0.26 to −0.11]). These findings were consistent among secondary outcomes (eg, incident rate ratio for all-cause return to hospital, 0.56 [95% CI, 0.43 to 0.73]) except for mortality, which was rare in both groups (incident rate ratio, 3.00 [95% CI, 0.42 to 21.22]). Discharge MAUD initiation was associated with a 51% decreased incidence of alcohol-related return to hospital (incident rate ratio, 0.49 [95% CI, 0.34 to 0.71]; absolute risk difference, −0.15 [95% CI, −0.22 to −0.09]).

**Conclusion and relevance:**

In this cohort study, discharge initiation of MAUD after alcohol-related hospitalization was associated with a large absolute reduction in return to hospital within 30 days. These findings support efforts to increase uptake of MAUD initiation at hospital discharge.

## Key Points

**Question:**

Is the initiation of medications for alcohol use disorder at hospital discharge associated with 30-day clinical outcomes among Medicare Part D beneficiaries?

**Findings:**

In this cohort study of 9834 alcohol-related hospitalizations, discharge initiation of medications for alcohol use disorder was associated with an absolute risk reduction of 18% in the composite outcome of return to hospital or death within 30 days compared with no initiation.

**Meaning:**

These findings support efforts to increase initiation of medications for alcohol use disorder at hospital discharge.

## Teaser

This cohort study investigates the association of medications for alcohol use disorder initiated at hospital discharge with return to hospital or death after alcohol-related hospitalizations.

## Introduction

In the US, alcohol use disorder (AUD) affects 29 million adults and imposes an annual economic burden of more than $250 billion.^1,2^ Excessive alcohol use is also the fourth leading cause of preventable mortality in the US, contributing to more than 140 000 annual deaths.^1^ Guideline-recommended treatment for AUD consists of behavioral interventions and medications for AUD (MAUD),^3^ including naltrexone, acamprosate, and disulfiram, which are approved by the Food and Drug Administration (FDA).^4^ Despite guideline recommendations, fewer than 2% of US adults with AUD received MAUD in 2019.^3,5^

Evidence supporting MAUD efficacy includes randomized clinical trials predominantly based in the outpatient setting with an end point of reduction in drinking.^6,7^ Oral naltrexone has been shown to reduce return to any drinking and heavy drinking, and acamprosate reduced return to any drinking among patients who achieved abstinence.^6,7^ Use of naltrexone has also been associated with reduced hospitalizations using observational data.^8^ Evidence supporting the efficacy of disulfiram is more limited, although it is recommended to use under specific circumstances, including patient preference or intolerance to naltrexone or acamprosate.^3^

Hospitalizations may serve as important touch points for AUD treatment engagement.^9^ Prescribing MAUD or referring patients to addiction treatment at discharge is a Joint Commission quality measure.^10^ However, postdischarge MAUD initiation among patients enrolled in Medicare rarely occurs.^11^ Reasons for underuse of MAUD are multifactorial and are in part associated with limited knowledge of effectiveness.^12,13^ To our knowledge, few studies have examined outcomes associated with MAUD initiation at hospital discharge.^14^ There were mixed results in 2 single-center, noncontrolled studies that examined preintervention vs postintervention 30-day readmission rates after implementation of interventions that increased naltrexone prescribing.^15,16^ Therefore, we applied a target trial emulation framework^17^ to investigate the association between discharge MAUD initiation and 30-day posthospitalization outcomes among Medicare beneficiaries hospitalized for alcohol-related conditions.

## Methods

We conducted a retrospective cohort study designed to emulate a randomized clinical trial of hospitalized patients with AUD (eTable 1 in Supplement 1). We used the 20% national sample of Centers for Medicare & Medicaid Services (CMS) administrative and pharmacy claims from 2015 to 2017. This study followed the Strengthening the Reporting of Observational Studies in Epidemiology (STROBE) reporting guideline. This project was approved by the CMS privacy board and the Beth Israel Deaconess Medical Center Institutional Review Board, which also waived informed consent due to the use of deidentified claims data.

### Population

The study sample included all persons who were continuously enrolled in Medicare Parts A, B, and D in the 12 months prior and 12 months after cohort entry or until death within 12 months after cohort entry. Beneficiaries enrolled in a Medicare Advantage plan at any point during the year were excluded due to unavailable administrative claims.

The cohort consisted of patients with acute care AUD hospitalizations in 2016 who were discharged to the community. AUD hospitalizations included those with a primary discharge diagnosis of AUD or a secondary diagnosis of AUD and a primary mental health discharge diagnosis. Given that the unit of analysis was unique hospitalization, patients could contribute multiple observations to the cohort. Mental health diagnoses were identified using the Healthcare Cost and Utilization Project Clinical Classification Software.^18^ AUD was defined using *International Statistical Classification of Diseases and Related Health Problems, Tenth Revision *(*ICD-10*) discharge diagnosis codes for alcohol abuse, dependence, and use (F10.1, F10.2, and F10.9), excluding “in remission” specifiers (F10.11, F10.21, and F10.91).^19^ To identify individuals newly receiving MAUD, patients with a pharmacy claim for naltrexone, acamprosate, or disulfiram within 90 days prior to hospitalization were excluded. Patients with liver disease and kidney failure (contraindications for naltrexone and acamprosate, respectively) were excluded. Liver disease and kidney failure were defined by discharge Elixhauser *ICD-10* codes and inpatient hemodialysis claims.^20^

### Outcomes

The primary outcome was a composite of all-cause mortality or return to hospital (including emergency department [ED] visits and readmissions [hospital or observation admissions]) within 30 days of discharge. Secondarily, we examined individual components of the composite outcome separately. We also investigated alcohol-related return to hospital; this was defined using primary or secondary *ICD-10* codes for acute alcohol-attributable diseases, which included previously mentioned AUD codes and other medical diagnoses related to alcohol (eTable 2 in Supplement 1).^21^ Finally, we examined outpatient primary care or mental health follow-up because these events are necessary for posthospitalization care transitions and ongoing AUD treatment (eTable 3 in Supplement 1).

### Exposure

The exposure of interest was discharge MAUD initiation, defined as a pharmacy claim for oral naltrexone, acamprosate, or disulfiram fills between the day before discharge and 2 days after discharge. Patients without discharge MAUD initiation were categorized as such regardless of subsequent MAUD fills. To avoid immortal time bias, we excluded patients who died or were readmitted prior to time zero, which was 2 days after hospital discharge.^22^

### Covariates

Covariates included sociodemographics, clinical factors, and hospitalization factors. Sociodemographic variables included age, sex, race and ethnicity, and census region. Race and ethnicity were self-reported and imputed using the Research Triangle Institute race code^23^ and were categorized as Hispanic of any race, non-Hispanic Black, non-Hispanic White, and other (including Asian, North American Native, and unknown, grouped due to small sample size). Race and ethnicity were included due to known disparities in AUD treatment. Medicaid dual eligibility was used to approximate lower income. County-level socioeconomic variables measured using the social deprivation index were included to account for socioeconomic disadvantage.^24^ We also included reason for entitlement to distinguish Medicare eligibility due to age vs disability. Patients with entitlement due to end-stage kidney disease (53 patients) were excluded because none received MAUD and thus a propensity score could not be estimated.

Clinical factors included medical and psychiatric comorbidities obtained from CMS Chronic Conditions Data Warehouse algorithms.^25^ Remote prior use of MAUD was defined using pharmacy claims for MAUD filled between 90 and 365 days prior to admission. We also included variables for health care use within the past year, including hospitalizations and ED visits. Alcohol withdrawal documented in any care setting (*ICD-10* codes F10.13, F10.23, and F10.93) and presence of alcohol-related comorbidities within the past year may correlate with more severe disease and so were also included (eTable 2 in Supplement 1). Behavioral treatment in the past 30 days and past year were identified using procedure codes for psychosocial and behavioral therapy as defined in prior literature.^26^

Hospitalization factors included an indicator variable for a primary discharge diagnosis of AUD, mood disorder, or other mental health diagnosis defined using Clinical Classification Software.^18^ The Elixhauser Comorbidity Index was used to measure comorbidity burden.^20^ A 3-level categorical variable for degree of psychiatric involvement in the hospitalization was created, comprising levels of psychiatric hospital, inpatient care by psychiatry or addiction medicine at a general hospital, or no psychiatry or addiction medicine involvement (eTable 4 in Supplement). Other hospitalization variables included self-directed discharge (defined by disposition of discharge against medical advice), length of hospital stay, and discharge month.

### Statistical Analysis

We applied propensity score matching to account for confounding by indication in associations between discharge MAUD initiation and posthospitalization outcomes. In our primary analysis, we estimated the propensity for receiving discharge MAUD initiation using aforementioned covariates in a logistic regression and used these propensity scores to perform a nearest-neighbor 3:1 match with replacement (caliper width, 0.2 of the SD of the logit of the propensity score).^27,28^ Covariate balance between groups was assessed using standard mean differences and propensity score distributions. We evaluated the association between discharge MAUD initiation and each outcome using modified Poisson regressions (Poisson regressions with robust variance) to estimate incident rate ratios (IRRs) and absolute risk differences (ARDs).^29^

Unadjusted outcomes were reported using modified Poisson regressions clustered by individual patients to account for repeated hospitalizations. We performed several sensitivity analyses to assess the robustness of our findings. First, because outcomes after 1 hospitalization may be affected by prior hospitalizations, we restricted our cohort to a single randomly selected hospitalization per patient and applied the same analytic approach. Second, because matching estimates treatment-associated outcomes among only the subset of patients who were matched, we applied overlap weighting, a method that weighs all observations in the cohort based on their probability of belonging to the opposite treatment group.^30^ We used overlap-weighted modified Poisson models with robust standard errors to account for clustering of repeated hospitalizations. Third, to increase comparability between treatment groups, we repeated the primary analysis using only the subgroup of patients with a primary AUD discharge diagnosis. Fourth, because patients who fill discharge medications may be more engaged in treatment compared with those who do not fill any medications, we restricted our cohort to patients who had at least 1 pharmacy fill for any medication within 2 days of discharge.

Finally, an E-value for the point estimate and CI was calculated to describe the minimum strength of association between treatment and outcome an unmeasured confounder would require in order to shift the effect size or CI to the null.^31^ Results were considered statistically significant when 95% CIs did not cross 1. All analyses were performed using Stata statistical software version 16.1 (StataCorp). Analysis was conducted in October 2022 to December 2023.

## Results

After excluding 2 hospitalizations that had an unspecified county code, we identified 9834 alcohol-related hospitalizations (median [IQR] age, 54 [46-62] years; 3205 hospitalizations among females [32.6%]; 1754 hospitalizations among Black [17.8%], 712 hospitalizations among Hispanic [7.2%], and 7060 hospitalizations among White [71.8%] individuals) representing 6794 unique individuals. Overall, 192 hospitalizations (2.0%) involved discharge MAUD initiation, including 112 hospitalizations (58.3%) with naltrexone, 53 hospitalizations (27.6%) with acamprosate, and 32 hospitalizations (16.7%) with disulfiram initiations; 9642 hospitalizations (98.0%) did not involve discharge MAUD. Prior to matching, a lower proportion of patients who received discharge MAUD initiation had prior psychosis (49 patients [25.5%] vs 3834 patients [39.8%]) and tobacco use disorder (98 patients [51.0%] vs 6402 patients [66.4%]) and a greater proportion had prior remote MAUD use (16 patients [8.3%] vs 171 patients [1.8%]) (Table 1). They were also more likely to have received care at psychiatric hospitals (85 patients [44.3%] vs 3225 patients [33.5%]) and by psychiatry or addiction medicine when hospitalized at general hospitals (65 patients [33.9%] vs 2477 patients [25.7%]). After matching, there was small residual imbalance in discharge month, but all other covariates were balanced, with standardized mean differences less than 0.1 (eFigure 1 in Supplement 1).

**Table 1.** Cohort Characteristics Before and After 3:1 Propensity Matching

Characteristic	Hospitalizations before matching, No. (%) (N = 9834)		SMD	Hospitalizations after matching, No. (%) (N = 768)		SMD
	Discharge MAUD (n = 192)	No discharge MAUD (n = 9642)		Discharge MAUD (n = 192)	No discharge MAUD (n = 576)	
Demographic						
Age, mean (SD), y	53.5 (12.5)	53.8 (12.6)	0.02	53.5 (12.5)	53.4 (13.0)	0.01
Sex						
Female	79 (41.1)	3126 (32.4)	0.18	79 (41.1)	225 (39.1)	0.04
Male	113 (58.9)	6516 (67.6)		113 (58.9)	351 (60.9)	
Race and ethnicity^a^						
Black	NR^c^	NR^c^	0.20	NR^c^	NR^c^	0.04
Hispanic	NR^c^	NR^c^		NR^c^	NR^c^	
White	NR^c^	NR^c^		NR^c^	NR^c^	
Other^b^	NR^c^	NR^c^		NR^c^	NR^c^	
Social deprivation index, mean (SD)	47.2 (26.1)	50.2 (28.2)	0.11	47.2 (26.1)	48.4 (27.3)	0.05
Region						
Northeast	59 (30.7)	2826 (29.3)	0.07	59 (30.7)	173 (30.0)	0.04
Midwest	42 (21.9)	2295 (23.8)		42 (21.9)	121 (21.0)	
South	69 (35.9)	3265 (33.9)		69 (35.9)	211 (36.6)	
West	22 (11.5)	1256 (13.0)		22 (11.5)	71 (12.3)	
Medicaid dual eligibility	135 (70.3)	7361 (76.3)	0.14	135 (70.3)	402 (69.8)	0.01
Reason for entitlement						
Age	31 (16.1)	1458 (15.1)	0.03	31 (16.1)	93 (16.1)	<0.01
Disability	161 (83.9)	8186 (84.9)		161 (83.9)	483 (83.9)	
Clinical						
Anxiety disorder	117 (60.9)	6236 (64.7)	0.08	117 (60.9)	354 (61.5)	0.01
Depression	180 (93.8)	8508 (88.2)	0.19	180 (93.8)	548 (95.1)	0.06
Posttraumatic stress disorder	36 (18.8)	1818 (18.9)	<0.01	36 (18.8)	100 (17.4)	0.04
Psychosis	49 (25.5)	3834 (39.8)	0.31	49 (25.5)	142 (24.7)	0.02
Bipolar disorder	79 (41.1)	4718 (48.9)	0.16	79 (41.1)	242 (42.0)	0.02
Tobacco use disorder	98 (51.0)	6402 (66.4)	0.32	98 (51.0)	294 (51.0)	<0.01
Opioid use disorder	44 (22.9)	2706 (28.1)	0.12	44 (22.9)	145 (25.2)	0.05
Liver disease	38 (19.8)	2776 (28.8)	0.21	38 (19.8)	103 (17.9)	0.05
Chronic kidney disease	19 (9.9)	2050 (21.3)	0.32	19 (9.9)	49 (8.5)	0.05
Prior alcohol withdrawal	64 (33.3)	2999 (31.1)	0.05	64 (33.3)	186 (32.3)	0.02
Alcohol-related comorbidity	20 (10.4)	1841 (19.1)	0.25	20 (10.4)	57 (9.9)	0.02
Remote MAUD use	16 (8.3)	171 (1.8)	0.30	16 (8.3)	42 (7.3)	0.04
Past-year primary care visit	116 (60.4)	5201 (53.9)	0.13	116 (60.4)	339 (58.9)	0.03
Past-year mental health visit	42 (21.9)	1964 (20.4)	0.04	42 (21.9)	118 (20.5)	0.03
Past-year hospitalization						
0	63 (32.8)	2287 (23.7)	0.31	63 (32.8)	175 (30.4)	0.06
1	43 (22.4)	1568 (16.3)		43 (22.4)	128 (22.2)	
>1	86 (44.8)	5787 (60.0)		86 (44.8)	273 (47.4)	
Past-year ED visit						
0	38 (19.8)	1768 (18.3)	0.08	38 (19.8)	109 (18.9)	0.02
1	36 (18.8)	1594 (16.5)		36 (18.8)	111 (19.3)	
>1	118 (61.5)	6281 (65.1)		118 (61.5)	356 (61.8)	
Past-year behavioral therapy	66 (34.4)	3091 (32.1)	0.05	66 (34.4)	204 (35.4)	0.02
Past 30-d behavioral therapy	19 (9.9)	1008 (10.5)	0.02	19 (9.9)	60 (10.4)	0.02
Hospitalization factor						
Discharge diagnosis category						
Alcohol	106 (55.2)	4763 (49.4)	0.35	106 (55.2)	315 (54.7)	0.04
Mood disorder	64 (33.3)	2534 (26.3)		64 (33.3)	201 (34.9)	
Other mental health	22 (11.5)	2347 (24.3)		22 (11.5)	60 (10.4)	
Elixhauser readmission index, mean (SD)	17.8 (12.5)	21.9 (13.2)	0.32	17.8 (12.5)	17.0 (11.7)	0.06
Inpatient addiction medicine or psychiatry						
None	42 (21.9)	3940 (40.9)	0.42	42 (21.9)	117 (20.3)	0.08
Psychiatric hospital	85 (44.3)	3225 (33.5)		85 (44.3)	278 (48.3)	
Psychiatry or addiction medicine at general hospital	65 (33.9)	2477 (25.7)		65 (33.9)	181 (31.4)	
Self-directed discharge	NR^c^	NR^c^	0.30	NR^c^	NR^c^	0.02
Length of stay, mean (SD), d	8.2 (6.8)	6.1 (6.1)	0.32	8.2 (6.8)	7.7 (8.2)	0.06
Discharge month	NR^c^	NR^c^	0.35	NR^c^	NR^c^	0.10

Abbreviations: MAUD, medications for alcohol use disorder (naltrexone, acamprosate, disulfiram); NR, not reported; SMD, standardized mean difference; ED, emergency department.

^a^ Self-reported and imputed race and ethnicity were determined using the Research Triangle Institute race code.^23^

^b^ Other includes Asian, North American Native, and unknown, grouped due to small sample sizes.

^c^ Redacted due to the Centers for Medicare & Medicaid Services cell suppression policy threshold for display of data (values <11 individuals).

### Primary Outcome

**Figure 1.**
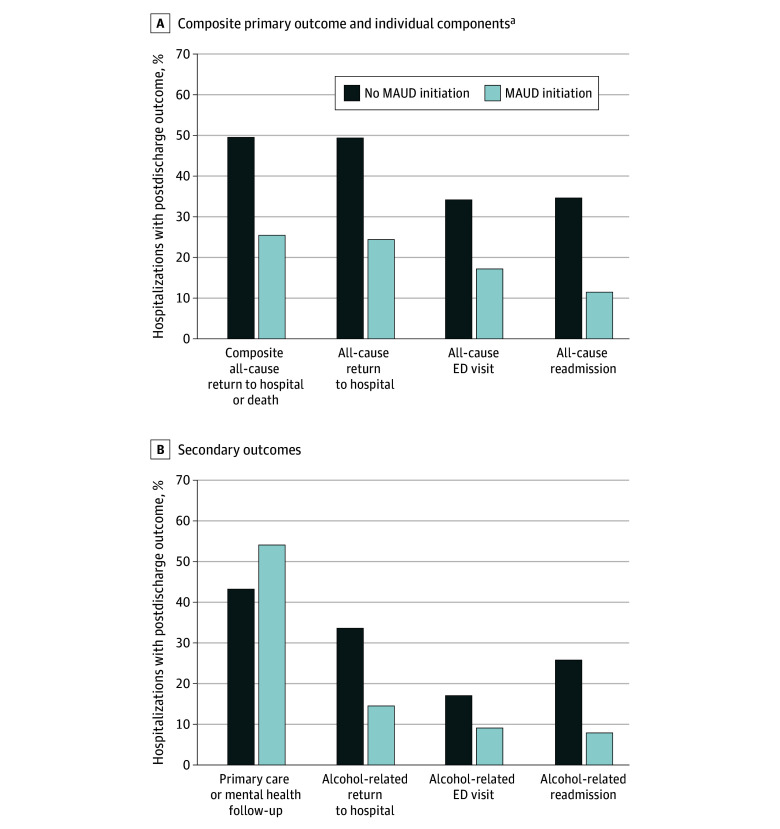
Unadjusted Posthospitalization Care Patterns After Alcohol-Related Hospitalizations at 30 d Return to hospital includes emergency department (ED) visits and hospital readmissions and observation stays. MAUD indicates medications for alcohol use disorder. ^a^Mortality not shown due to the Centers for Medicare & Medicaid Services cell suppression policy threshold for display of data (values <11 individuals).

The primary outcome occurred among 4843 hospitalizations (49.3%), including 49 hospitalizations (25.5%) that involved discharge MAUD initiation and 4794 hospitalizations (49.7%) that did not (Figure 1A). This was mostly contributed by 3340 all-cause ED visits (34.0%) and 3375 all-cause readmissions (34.3%) within 30 days. There were 97 deaths (1.0%) within 30 days of discharge. After matching, discharge MAUD initiation was associated with a 42% decreased incidence of the primary outcome (IRR, 0.58 [95% CI, 0.45 to 0.76]; ARD, −0.18 [95% CI, −0.26 to −0.11]) (Table 2).

**Table 2.** Association Between Discharge Initiation of MAUD and 30-d Composite Primary Outcome

Outcome	Hospitalizations, No. (%) (N = 9834)		Unadjusted IRR (95% CI)	Adjusted IRR (95% CI)	Adjusted ARD (95% CI)
	Discharge MAUD initiation (n = 192)	No discharge MAUD initiation (n = 9642)			
Primary composite outcome					
Primary composite outcome^a^^,^^b^	49 (25.5)	4794 (49.7)	0.51 (0.40 to 0.65)	0.58 (0.45 to 0.76)	−0.18 (−0.26 to −0.11)
Individual component					
All-cause return to hospital^b^	47 (24.5)	4770 (49.5)	0.49 (0.39 to 0.64)	0.56 (0.43 to 0.73)	−0.19 (−0.27 to −0.12)
All-cause ED visits	33 (17.2)	3307 (34.3)	0.50 (0.37 to 0.68)	0.57 (0.41 to 0.80)	−0.13 (−0.20 to −0.06)
All-cause readmissions	22 (11.5)	3353 (34.8)	0.33 (0.22 to 0.49)	0.42 (0.27 to 0.63)	−0.16 (−0.22 to −0.10)
Mortality	NR^c^	NR^c^	1.05 (0.26 to 4.26)	3.00 (0.42 to 21.22)	0.01 (−0.01 to 0.02)

Abbreviations: ARD, absolute risk difference; ED, emergency department; IRR, incident rate ratio; NR, not reported; MAUD, medications for alcohol use disorder.

^a^ All-cause return to hospital or mortality.

^b^ Return to hospital includes ED visits and hospital readmissions.

^c^ Redacted values due to the Centers for Medicare & Medicaid Services cell suppression policy threshold for display of data (values <11 individuals).

### Secondary Outcomes

Unadjusted secondary outcomes are shown in Figure 1B. In adjusted models, discharge MAUD initiation was associated with a decreased incidence of all-cause ED visits (IRR, 0.57 [95% CI, 0.41 to 0.80]; ARD, −0.13 [95% CI, −0.20 to −0.06]) and all-cause readmissions (IRR, 0.42 [95% CI, 0.27 to 0.63]; ARD, −0.16 [95% CI, −0.22 to −0.10]), but there was no significant difference in mortality (IRR, 3.00 [95% CI, 0.42 to 21.22]; ARD, 0.01 [95% CI, −0.01 to 0.02]). Discharge MAUD initiation was also associated with decreased incidence of all-cause return to hospital (IRR, 0.56 [95% CI, 0.43 to 0.73]; ARD, −0.19 [95% CI, −0.27 to −0.12]) (Table 2) and alcohol-related return to hospital (IRR, 0.49 [95% CI, 0.34 to 0.71]; ARD, −0.15 [95% CI, −0.22 to −0.09), which was mostly contributed by alcohol-related readmissions (Table 3). Alcohol-related ED visits did not reach statistical significance in relative terms (IRR, 0.61 [95% CI, 0.37 to 1.01]; ARD, −0.05 [95% CI, −0.11 to −0.001). Overall, 4290 hospitalizations (43.6%) involved 30-day primary care or mental health follow-up, and discharge MAUD initiation was associated with increased incidence of this outcome (IRR, 1.22 [95% CI, 1.04 to 1.44]; ARD, 0.10 [95% CI, 0.02 to 0.18]).

**Table 3.** Association Between Discharge Initiation of MAUD and 30-d Secondary Outcomes

Outcome	Hospitalizations, No. (%) (N = 9834)		Unadjusted IRR (95% CI)	Adjusted IRR (95% CI)	Adjusted ARD (95% CI)
	Discharge MAUD initiation (n = 192)	No discharge MAUD initiation (n = 9642)			
Alcohol-related return to hospital^a^	28 (14.6)	3247 (33.7)	0.43 (0.31 to 0.61)	0.49 (0.34 to 0.71)	−0.15 (−0.22 to −0.09)
Alcohol-related ED visits	17 (8.9)	1635 (17.0)	0.52 (0.33 to 0.83)	0.61 (0.37 to 1.01)	−0.05 (−0.11 to −0.001)
Alcohol-related readmissions	15 (7.8)	2493 (25.9)	0.30 (0.19 to 0.49)	0.36 (0.21 to 0.59)	−0.14 (−0.19 to −0.09)
Primary care or mental health follow-up	104 (54.2)	4186 (43.4)	1.25 (1.09 to 1.42)	1.22 (1.04 to 1.44)	0.10 (0.02 to 0.18)

Abbreviations: ARD, absolute risk difference; ED, emergency department; IRR, incident rate ratio; MAUD, medications for alcohol use disorder.

^a^ Return to hospital includes ED visits and hospital readmission.

### Sensitivity Analyses

**Figure 2.**
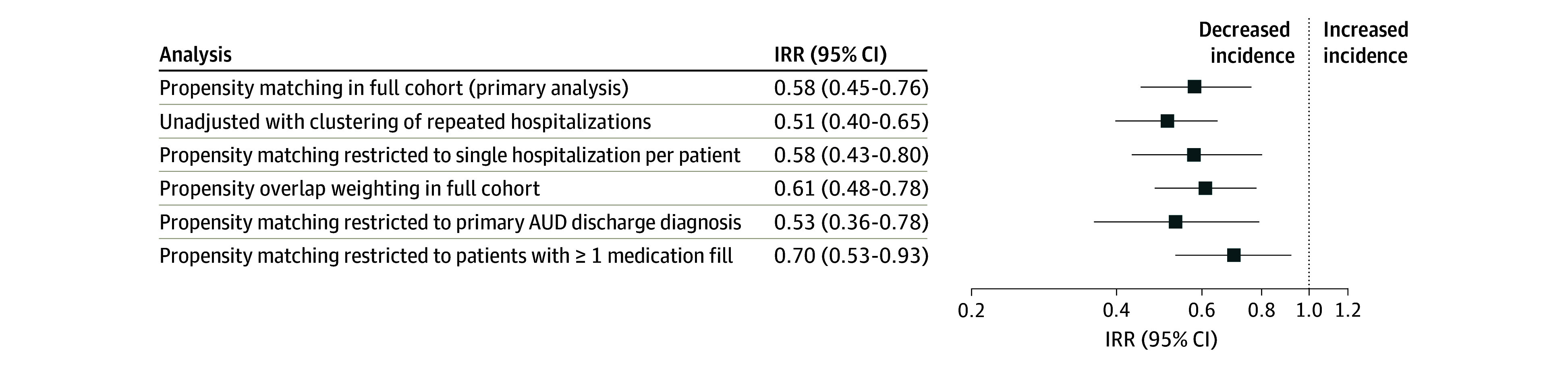
Association Between Discharge Initiation and Primary Outcome in Sensitivity Analyses Associations are shown between discharge initiation of medications for alcohol use disorder and the primary outcome (composite 30-day all-cause return to hospital or mortality). AUD indicates alcohol use disorder; IRR, incident rate ratio.

Sensitivity analyses yielded consistent point estimates and statistical significance for the association between discharge MAUD initiation and the primary outcome (Figure 2). In the analysis restricted to a single hospitalization per patient among 664 patients, the IRR was 0.58 (95% CI, 0.43-0.80), although there were postmatching residual imbalances in discharge month and a greater proportion of patients with more than 1 past-year ED visit in the group that did not receive MAUD (eTable 5 and eFigure 2 in Supplement 1). In the analysis using propensity overlap weighting among 9834 patients, all covariates were balanced on the mean (eTable 6 in Supplement 1) and the IRR was 0.61 (95% CI, 0.48, 0.78). In the analysis restricted to 304 patients with a primary AUD discharge diagnosis, there were residual imbalances in discharge month; there was a higher social deprivation index and more frequent anxiety and depression in the group that did not receive MAUD (eTable 7 and eFigure 3 in Supplement 1), and the IRR was 0.53 (95% CI, 0.36-0.78). In the analysis restricted to 768 patients who had at least 1 medication fill, there were residual imbalances in race and ethnicity and discharge month (eTable 8 and eFigure 4 in Supplement 1); the IRR was 0.70 (95% CI, 0.53-0.93). The E-value for the primary analysis was 2.84 for the point estimate and 1.96 for the CI.

## Discussion

In this cohort study of a national sample of Medicare Part D beneficiaries with alcohol-related hospitalizations in 2016, discharge MAUD initiation was associated with a 42% relative and 18% absolute reduction in 30-day all-cause mortality or return to hospital. Nearly half of hospitalizations were followed by 30-day mortality or return to hospital, and less than half involved primary care or mental health follow-up. Given the established benefit of providing brief interventions to hospitalized patients with AUD^32^ and the efficacy of MAUD in other care settings,^6^ our findings highlight the potential benefit of interventions to promote behavior change through initiation of MAUD after alcohol-related hospitalizations.

Our study builds on prior work characterizing clinical outcomes after alcohol-related hospitalizations. We found a higher 30-day readmission rate (34.3%) than a prior study that identified a 14% rate after alcohol-related hospitalizations in 2015.^33^ These differences may be explained by our cohort characteristics, including older age and a high proportion of patients with disabilities. The high rate of return to hospital in the 30 days after alcohol-related hospitalizations and the finding that less than half of such patients attended a primary care or mental health follow-up appointment emphasizes an urgent need to implement interventions to promote more effective transitions of care to the outpatient setting.

Our study also builds on prior single-center studies of MAUD initiation in hospital settings. Wei et al^15^ performed a preimplementation vs postimplementation study of a discharge-planning tool that increased naltrexone prescribing from 0% to 64% and found an absolute reduction in the rate of 30-day ED visits and readmissions (13% and 15%, respectively). We found a similar absolute risk difference (13% and 16%). Stevens et al^16^ performed a similar preintervention vs postintervention evaluation of an intervention that increased naltrexone prescribing from 2% to 28% for patients admitted with alcohol detoxification or withdrawal through clinician education and dissemination of a decision support tool. This study found a reduction in 30-day ED visits but not readmissions. Although more than 20% of readmissions occur at different hospitals, neither prior study evaluated health care use at other health systems.^34^ An open-label pilot trial^35^ found a 48% relative reduction in 30-day readmissions among patients randomized to injectable naltrexone compared with linkage to care alone. These underpowered results did not reach statistical significance but reflect a similar magnitude of effect size as observed in our study. Our findings add to the existing literature of clinical effectiveness of MAUD in hospital discharge by showing that MAUD initiation was associated with improved posthospitalization outcomes in a national sample.

One component of interventions to promote AUD treatment engagement in the discharge and posthospitalization setting should include increased MAUD prescribing. Known barriers for prescribing in the inpatient setting include clinician knowledge gaps, concern about discharge follow-up, lack of institutional prioritization, and stigma of AUD.^13^ However, prior implementation efforts around AUD hospitalizations have shown promise.^15,16^ Expansion of existing programs to promote clinician training through video conferencing^36^ and pharmacist-led posthospitalization transition care^37^ may also prove beneficial in expanding treatment access. In addition, interventions should include facilitation of outpatient follow-up, which is needed for monitoring or initiating MAUD (if it did not already occur at discharge), providing counseling, and referring patients to additional behavioral health services.

### Limitations

This study has several limitations. There are inherent limitations of this observational study design, including unmeasured confounding.^17^ The E-value indicates that an unobserved confounder would need to have an IRR of 2.84 to shift our effect size to the null. For example, we were unable to account for healthy user bias or psychosocial factors, including patient motivation. Notably, we observed a large effect size after controlling for multiple observed confounders. The effect size was attenuated in the analysis restricted to patients who had at least 1 discharge medication fill, indicating the presence of some residual confounding, but the effect size remained large and retained statistical significance. Still, our results likely represent upper bound estimates, and a randomized trial is needed to answer this question definitively. Results may not be generalizable to patients who are younger, do not have disabilities, or are Medicare Advantage beneficiaries. Diagnosis codes for case identification of AUD are unable to discern severity of disease, although we were able to use variables for prior withdrawal and alcohol-related health care use as surrogates for severity. Owing to bundled inpatient billing, we were unable to identify rates of long-acting injectable naltrexone that was initiated during hospitalization. However, injectable naltrexone is the least used FDA-approved MAUD,^38,39^ and many hospitals may not have it on formulary.^40^ We were also unable to identify use of nonpharmacologic treatment, including 12-step facilitation or behavioral interventions. Additionally, data from hospitalizations in 2016 may not be representative of current AUD outcomes.

## Conclusions

In this retrospective cohort study of Medicare Part D beneficiaries with alcohol-related hospitalizations, return to hospital within 30 days was common and initiation of MAUD at discharge was associated with a large reduction in return to hospital within 30 days. These findings support ongoing efforts to increase access to MAUD in the posthospitalization setting.

## Notes

Supplement 1. **eTable 1.** Components of a Theoretical Target Trial and Emulation **eTable 2.** Identification of Alcohol-Related Disease **eTable 3.** Identification of Primary Care or Mental Health Follow-Up **eTable 4.** Identification of Addiction Medicine or Psychiatry Involvement During Hospitalization **eTable 5.** Characteristics Before and After Propensity Matching in Sensitivity Analysis Restricted to Single Hospitalization per Patient **eTable 6.** Characteristics Before and After Weighting in Sensitivity Analysis Using Overlap Propensity Weighting **eTable 7.** Characteristics Before and After Propensity Matching in Sensitivity Analysis Restricted to Primary Alcohol Use Disorder Discharge Diagnosis **eTable 8.** Characteristics Before and After Propensity Matching in Sensitivity Analysis Restricted to Patients With >1 Discharge Medication Fill **eFigure 1.** Propensity Score Distribution Before and After 3:1 Matching in Primary Analysis **eFigure 2.** Propensity Score Distribution Before and After 3:1 Matching in Sensitivity Analysis Restricted to a Single Hospitalization per Patient **eFigure 3.** Propensity Score Distribution Before and After 3:1 Matching in Sensitivity Analysis Restricted to Primary Alcohol Use Disorder Discharge Diagnoses **eFigure 4.** Propensity Score Distribution Before and After 3:1 Matching in Sensitivity Analysis Restricted to Patients With >1 Discharge Medication Fill

Supplement 2.
Data Sharing Statement
