# Supplementary material for: Outcomes After Initiation of Medications for Alcohol Use Disorder at Hospital Discharge
Source: JAMA Netw Open. 2024 Mar 29;7(3):e243387. doi: 10.1001/jamanetworkopen.2024.3387 (PMC10980961; doi:10.1001/jamanetworkopen.2024.3387)
Supplement: Supplement 1. — eTable 1. Components of a Theoretical Target Trial and Emulation eTable 2. Identification of Alcohol-Related Disease eTable 3. Identification of Primary Care or Mental Health Follow-Up eTable 4. Identification of Addiction Medicine or Psychiatry Involvement During Hospitalization eTable 5. Characteristics Before and After Propensity Matching in Sensitivity Analysis Restricted to Single Hospitalization per Patient eTable 6. Characteristics Before and After Weighting in Sensitivity Analysis Using Overlap Propensity Weighting eTable 7. Characteristics Before and After Propensity Matching in Sensitivity Analysis Restricted to Primary Alcohol Use Disorder Discharge Diagnosis eTable 8. Characteristics Before and After Propensity Matching in Sensitivity Analysis Restricted to Patients With >1 Discharge Medication Fill eFigure 1. Propensity Score Distribution Before and After 3:1 Matching in Primary Analysis eFigure 2. Propensity Score Distribution Before and After 3:1 Matching in Sensitivity Analysis Restricted to a Single Hospitalization per Patient eFigure 3. Propensity Score Distribution Before and After 3:1 Matching in Sensitivity Analysis Restricted to Primary Alcohol Use Disorder Discharge Diagnoses eFigure 4. Propensity Score Distribution Before and After 3:1 Matching in Sensitivity Analysis Restricted to Patients With >1 Discharge Medication Fill [file jamanetwopen-e243387-s001.pdf]

Supplemental Online Content

Bernstein EY, Baggett TP, Trivedi S, Herzig SJ, Anderson TS. Outcomes after initiation of medications for alcohol use disorder at hospital discharge. *JAMA Netw Open*. 2024;7(3):e243387. doi:10.1001/jamanetworkopen.2024.3387

eTable 1. Components of a Theoretical Target Trial and Emulation

eTable 2. Identification of Alcohol-Related Disease

eTable 3. Identification of Primary Care or Mental Health Follow-Up

eTable 4. Identification of Addiction Medicine or Psychiatry Involvement During Hospitalization

eTable 5. Characteristics Before and After Propensity Matching in Sensitivity Analysis Restricted to Single Hospitalization per Patient

eTable 6. Characteristics Before and After Weighting in Sensitivity Analysis Using Overlap Propensity Weighting

eTable 7. Characteristics Before and After Propensity Matching in Sensitivity Analysis Restricted to Primary Alcohol Use Disorder Discharge Diagnosis

eTable 8. Characteristics Before and After Propensity Matching in Sensitivity Analysis Restricted to Patients With >1 Discharge Medication Fill

eFigure 1. Propensity Score Distribution Before and After 3:1 Matching in Primary Analysis

eFigure 2. Propensity Score Distribution Before and After 3:1 Matching in Sensitivity Analysis Restricted to a Single Hospitalization per Patient

eFigure 3. Propensity Score Distribution Before and After 3:1 Matching in Sensitivity Analysis Restricted to Primary Alcohol Use Disorder Discharge Diagnoses

eFigure 4. Propensity Score Distribution Before and After 3:1 Matching in Sensitivity Analysis Restricted to Patients With >1 Discharge Medication Fill

This supplemental material has been provided by the authors to give readers additional information about their work.

© 2024 Bernstein EY et al. *JAMA Netw Open.*

Supplemental Table 1. Components of a theoretical target trial and emulation

**Protocol component**	**Target trial**	**Emulation**
Eligibility criteria	Inclusion criteria:<br>1. Patients hospitalized due to alcohol use disorder (AUD)<br><br>Exclusion criteria:<br>1. Use of medications for AUD (MAUD) in 90 days prior to hospitalization<br>2. Contraindications to MAUD (liver disease and renal failure)	Inclusion criteria:<br>1. AUD hospitalization defined using discharge diagnosis ICD codes<br><br>Exclusion criteria:<br>1. MAUD fill in 90 days prior to hospitalization<br>2. Contraindications to MAUD defined using hemodialysis claims and ICD codes
Treatment strategies	Initiation MAUD vs no MAUD on hospital discharge	Initiation of MAUD, defined by pharmacy fill within 2 days of discharge vs no MAUD on hospital discharge
Assignment procedures	Randomization of patients during hospitalization	Ensure comparability of treatment groups by adjusting for observed baseline confounders using a 1:3 match based on propensity scores.
Follow-up period	Day of hospital discharge to 30 days after discharge	Two days after hospital discharge (to allow time for medication fills) to 30 days after discharge.
Outcomes	Primary outcome: Composite of all-cause return to hospital or mortality<br><br>Secondary outcomes: All-cause emergency department visits, all-cause hospital readmissions, mortality, return to hospital for alcohol-related diagnoses, and primary care or mental health follow-up	Same as target trial
Causal contrast of interest	Intention-to-treat	Same as target trial
Analysis plan	Intention-to-treat analysis to compare outcomes between groups assigned to each treatment strategy	Variables from the baseline and pre-exposure lookback period were used to generate propensity scores. A 3:1 propensity match was used to emulate randomization of assignment to treatment strategies. Biases were assessed through several sensitivity analyses including restriction of the cohort to allow for greater similarity between treatment groups. Unmeasured residual confounding was assessed by measuring an E-value.

© 2024 Bernstein EY et al. *JAMA Netw Open.*

Supplemental Table 2. Identification of alcohol-related disease

**Diagnosis**	**ICD-10 code**
Alcohol use/abuse/dependence[A]	F10.1x, F10.2x, F10.9x excluding "in remission" specifiers (F10.11, F10.21, F10.91)
Alcohol-related cardiomyopathy	I42.6
Alcohol-related polyneuropathy	G62.1
Alcohol-related gastritis	K29.2
Alcohol-related liver disease	K70.0-K70.4, K70.9
Alcohol-related myopathy	G72.1
Alcohol-related psychosis	F10.15, F10.25, F10.95
Alcohol-induced acute pancreatitis	K85.2
Alcohol-induced chronic pancreatitis	K86.0
Degeneration of nervous system due to alcohol	G31.2

[A]These diagnosis codes were used only for the alcohol-related return to hospital outcome and not the alcohol-related comorbidity covariate.

Supplemental Table 3. Identification of primary care or mental health follow-up

Step	Criteria
Outpatient office visit	CPT/HCPCS codes within 30 days of discharge: 99201 to 99215, 99241 to 99245, 99381 to 99387, 99391 to 99397, G0438 to G0439, and 99495 to 99496
Primary care visit	Provider specialty codes: general practice (01), family practice (08), internal medicine (11), osteopathic medicine (12), general medicine (38), preventive medicine (84), nurse practitioner (50), physician assistant (97)
Mental health visit	Provider specialty codes: psychiatry (26), geriatric psychiatry (27), addiction medicine (79), psychologist (62), clinical psychologist (68)

© 2024 Bernstein EY et al. *JAMA Netw Open.*

Supplemental Table 4. Identification of addiction medicine or psychiatry involvement during hospitalization

Category	Criteria
Psychiatric hospital	CMS psychiatric special unit code (S)
Psychiatry or addiction medicine at a general hospital	Not psychiatric hospital with presence of inpatient billing by provider specialty codes for psychiatry (26), geriatric psychiatry (27), or addiction medicine (79)
No psychiatry or addiction medicine involvement	Does not meet either of the criteria above

© 2024 Bernstein EY et al. *JAMA Netw Open.*

Supplemental Table 5. Characteristics before and after propensity matching in sensitivity analysis restricted to single hospitalization per patient

Characteristic	Before matching			After matching		
	Discharge MAUD (N=166)	No discharge MAUD (N=6,628)	SMD	Discharge MAUD (N=166)	No discharge MAUD (N=498)	SMD
**Demographics**						
Age, mean (SD), y	53.8 (12.5)	54.5 (13.1)	0.06	53.8 (12.5)	53.4 (13.8)	0.03
Sex, n (%)						
Female	67 (40.4)	2276 (34.3)	0.12	67 (40.4)	191 (38.4)	0.04
Male	99 (59.6)	4352 (65.7)		99 (59.6)	307 (61.6)	
Race/ethnicity, n (%)[A]						
Black	*	*	0.20	*	*	0.09
Hispanic	*	*		*	*	
White	*	*		*	*	
Other	*	*		*	*	
Social Deprivation Index, mean (SD)	47.7 (25.3)	50.2 (28.3)	0.09	47.7 (25.3)	47.9 (27.9)	0.01
Region, n (%)						
Northeast	51 (30.7)	1827 (27.6)	0.09	51 (30.7)	150 (30.1)	0.06
Midwest	38 (22.9)	1600 (24.1)		38 (22.9)	109 (21.9)	
South	58 (34.9)	2291 (34.6)		58 (34.9)	172 (34.5)	
West	19 (11.4)	910 (13.7)		19 (11.4)	67 (13.5)	
Medicaid dual-eligibility, n (%)	115 (69.3)	4883 (73.7)	0.10	115 (69.3)	350 (70.3)	0.02
Reason for entitlement, n (%)						
Age	28 (16.9)	1148 (17.3)	0.01	28 (16.9)	69 (13.9)	0.08
Disability	138 (83.1)	5480 (82.7)		138 (83.1)	429 (86.1)	
**Clinical factors,** n (%)						
Anxiety disorder	96 (57.8)	3875 (58.5)	0.01	96 (57.8)	273 (54.8)	0.06
Depression	155 (93.4)	5661 (85.4)	0.26	155 (93.4)	471 (94.6)	0.05
Posttraumatic stress disorder	29 (17.5)	994 (15.0)	0.07	29 (17.5)	78 (15.7)	0.05
Psychosis	37 (22.3)	2275 (34.3)	0.27	37 (22.3)	109 (21.9)	0.01
Bipolar disorder	67 (40.4)	2802 (42.3)	0.04	67 (40.4)	216 (43.4)	0.06
Tobacco use disorder	80 (48.2)	4023 (60.7)	0.25	80 (48.2)	250 (50.2)	0.04
Opioid use disorder	34 (20.5)	1552 (23.4)	0.07	34 (20.5)	106 (21.3)	0.02
Liver disease	28 (16.9)	1617 (24.4)	0.19	28 (16.9)	92 (18.5)	0.04
Chronic kidney disease	14 (8.4)	1269 (19.1)	0.31	14 (8.4)	45 (9.0)	0.02

© 2024 Bernstein EY et al. *JAMA Netw Open.*

Prior alcohol withdrawal	46 (27.7)	1391 (21.0)	0.16	46 (27.7)	144 (28.9)	0.03
Alcohol-related comorbidities	13 (7.8)	894 (13.5)	0.18	13 (7.8)	42 (8.4)	0.02
Remote MAUD use	*	*	0.19	*	*	0.02
Past-year primary care visit	98 (59.0)	3640 (54.9)	0.08	98 (59.0)	304 (61.0)	0.04
Past-year mental health visit	32 (19.3)	1278 (19.3)	<0.01	32 (19.3)	115 (23.1)	0.09
Past-year hospitalizations						
None	63 (38.0)	2106 (31.8)		63 (38.0)	190 (38.2)	
One	38 (22.9)	1278 (19.3)	0.20	38 (22.9)	110 (22.1)	0.02
Greater than 1	65 (39.2)	3244 (48.9)		65 (39.2)	198 (39.8)	
Past-year ED visits						
None	36 (21.7)	1485 (22.4)		36 (21.7)	97 (19.5)	
One	33 (19.9)	1265 (19.1)	0.02	33 (19.9)	82 (16.5)	0.12
Greater than 1	97 (58.4)	3878 (58.5)		97 (58.4)	319 (64.1)	
Past-year behavioral therapy	49 (29.5)	1837 (27.7)	0.04	49 (29.5)	136 (27.3)	0.05
Past-30-day behavioral therapy	13 (7.8)	639 (9.6)	0.06	13 (7.8)	33 (6.6)	0.05
**Hospitalization factors**						
Discharge diagnosis category, n (%)						
Alcohol	94 (56.6)	3141 (47.4)		94 (56.6)	276 (55.4)	
Mood disorder	55 (33.1)	1772 (26.7)	0.42	55 (33.1)	176 (35.3)	0.05
Other mental health	17 (10.2)	1715 (25.9)		17 (10.2)	46 (9.2)	
Elixhauser readmission index, mean (SD)	18.0 (12.7)	22.1 (13.2)	0.32	18.0 (12.7)	18.8 (12.1)	0.07
Inpatient addiction medicine or psychiatry, n (%)						
None	39 (23.5)	2697 (40.7)		39 (23.5)	114 (22.9)	
Psychiatric hospital	72 (43.4)	2294 (34.6)	0.38	72 (43.4)	226 (45.4)	0.04
Psychiatry/addiction medicine at general hospital	55 (33.1)	1637 (24.7)		55 (33.1)	158 (31.7)	
Self-directed discharge, n (%)	*	*	0.27	*	*	<0.01
Length of stay, mean (SD)	7.8 (6.0)	6.2 (6.3)	0.26	7.8 (6.0)	8.0 (9.8)	0.02
Discharge month	*	*	0.33	*	*	0.14

MAUD = Medications for alcohol use disorder (naltrexone, acamprosate, disulfiram), SMD = Standardized mean difference, SD = Standard deviation, ED = Emergency department
*Indicates redacted due to CMS cell suppression policy threshold for display of data (values <11)
[A] Self-reported and imputed race and ethnicity were determined using the Research Triangle Institute race code. Other includes Asian, North American Native, and unknown, grouped due to small sample size.

© 2024 Bernstein EY et al. *JAMA Netw Open.*

Supplemental Table 6. Characteristics before and after weighting in sensitivity analysis using overlap propensity weighting

Characteristic	Before weighting			After weighting		
	Discharge MAUD (N=192)	No discharge MAUD (N=9,642)	SMD	Discharge MAUD (N=192)	No discharge MAUD (N=9,642)	SMD
**Demographics**						
Age, mean (SD), y	53.5 (12.5)	53.8 (12.6)	0.02	53.6 (2.5)	53.6 (18.3)	<0.01
Sex, %						
Female	41.1	32.4	0.18	41.0	41.0	<0.01
Male	58.9	67.6		59.0	59.0	
Race/ethnicity, %						
Black	*	*	0.20	*	*	<0.01
Hispanic	*	*		*	*	
White	*	*		*	*	
Other	*	*		*	*	
Social Deprivation Index, mean (SD)	47.2 (26.1)	50.2 (28.2)	0.11	47.2 (5.1)	47.2 (38.5)	<0.01
Region, %						
Northeast	30.7	29.3	0.07	30.5	30.5	<0.01
Midwest	21.9	23.8		22.2	22.2	
South	35.9	33.9		35.7	35.7	
West	11.5	13.0		11.6	11.6	
Medicaid dual-eligibility, %	70.3	76.3	0.14	70.3	70.3	<0.01
Reason for entitlement, %						
Age	16.1	15.1	0.03	16.1	16.1	<0.01
Disability	83.9	84.9		83.9	83.9	
**Clinical factors, %**						
Anxiety disorder	60.9	64.7	0.08	61.2	61.2	<0.01
Depression	93.8	88.2	0.19	93.7	93.7	<0.01
Posttraumatic stress disorder	18.8	18.9	<0.01	18.5	18.5	<0.01
Psychosis	25.5	39.8	0.31	25.8	25.8	<0.01
Bipolar disorder	41.1	48.9	0.16	41.6	41.6	<0.01
Tobacco use disorder	51.0	66.4	0.32	51.8	51.8	<0.01
Opioid use disorder	22.9	28.1	0.12	23.0	23.0	<0.01
Liver disease	19.8	28.8	0.21	20.3	20.3	<0.01
Chronic kidney disease	9.9	21.3	0.32	10.0	10.0	<0.01
Prior alcohol withdrawal	33.3	31.1	0.05	33.0	33.0	<0.01

© 2024 Bernstein EY et al. *JAMA Netw Open.*

Alcohol-related comorbidities	10.4	19.1	0.25	10.7	10.7	<0.01
Remote MAUD use	8.3	1.8	0.30	7.9	7.9	<0.01
Past-year primary care visit	60.4	53.9	0.13	60.1	60.1	<0.01
Past-year mental health visit	21.9	20.4	0.04	21.7	21.7	<0.01
Past-year hospitalizations						
None	32.8	23.7		32.6	32.6	
One	22.4	16.3	0.31	22.4	22.4	<0.01
Greater than 1	44.8	60.0		45.0	45.0	
Past-year ED visits						
None	19.8	18.3		19.9	19.9	
One	18.8	16.5	0.08	18.8	18.8	<0.01
Greater than 1	61.5	65.1		61.2	61.2	
Past-year behavioral therapy	34.4	32.1	0.05	34.0	34.0	<0.01
Past-30-day behavioral therapy	9.9	10.5	0.02	10.0	10.0	<0.01
**Hospitalization factors**						
Discharge diagnosis category, %						
Alcohol	55.2	49.4		54.7	54.7	
Mood disorder	33.3	26.3	0.35	33.5	33.5	<0.01
Other mental health	11.5	24.3		11.7	11.7	
Elixhauser readmission index, mean (SD)	17.8 (12.5)	21.9 (13.2)	0.32	17.9 (2.5)	17.9 (16.7)	<0.01
Inpatient addiction medicine or psychiatry, %						
None	21.9	40.9		22.4	22.4	
Psychiatric hospital	44.3	33.5	0.42	43.8	43.8	<0.01
Psychiatry/addiction medicine at general hospital	33.9	25.7		33.8	33.8	
Self-directed discharge, %	*	*	0.30	*	*	<0.01
Length of stay, mean (SD)	8.2 (6.8)	6.1 (6.0)	0.32	8.1 (1.3)	8.1 (14.6)	<0.01
Discharge month	*	*	0.35	*	*	<0.01

MAUD = Medications for alcohol use disorder (naltrexone, acamprosate, disulfiram), SMD = Standardized mean difference, SD = Standard deviation, ED = Emergency department
*Indicates redacted due to CMS cell suppression policy threshold for display of data (values <11)
[A] Self-reported and imputed race and ethnicity were determined using the Research Triangle Institute race code. Other includes Asian, North American Native, and unknown, grouped due to small sample size.

© 2024 Bernstein EY et al. *JAMA Netw Open.*

Supplemental Table 7. Characteristics before and after propensity matching in sensitivity analysis restricted to primary AUD discharge diagnosis

Characteristic	Before matching			After matching		
	**Discharge MAUD (N=106)**	**No discharge MAUD (N=4,763)**	**SMD**	**Discharge MAUD (N=76)**	**No discharge MAUD (N=228)**	**SMD**
**Demographics**						
Age, mean (SD), y	56.3 (11.2)	57.4 (11.6)	0.09	56.3 (11.2)	56.4 (12.1)	<0.01
Sex, n (%)						
Female	37 (34.9)	1465 (30.8)	0.09	37 (34.9)	113 (35.5)	0.01
Male	69 (65.1)	3298 (69.2)		69 (65.1)	205 (64.5)	
Race/ethnicity, n (%)[A]						
Black	*	*	0.26	*	*	0.06
Hispanic	*	*		*	*	
White	*	*		*	*	
Other	*	*		*	*	
Social Deprivation Index, mean (SD)	49.1 (26.0)	49.5 (28.2)	0.02	49.1 (26.0)	51.7 (27.9)	0.10
Region, n (%)						
Northeast	*	*	0.15	*	*	0.05
Midwest	*	*		*	*	
South	*	*		*	*	
West	*	*		*	*	
Medicaid dual-eligibility, n (%)	74 (69.8)	3314 (69.6)	0.01	74 (69.8)	219 (68.9)	0.02
Reason for entitlement, n (%)						
Age	21 (19.8)	1065 (22.4)	0.06	21 (19.8)	61 (19.2)	0.02
Disability	85 (80.2)	3698 (77.6)		85 (80.2)	257 (80.8)	
**Clinical factors,** n (%)						
Anxiety disorder	62 (58.5)	2798 (58.7)	0.01	62 (58.5)	204 (64.2)	0.12
Depression	96 (90.6)	3896 (81.8)	0.26	96 (90.6)	299 (94.0)	0.13
Posttraumatic stress disorder	19 (17.9)	725 (15.2)	0.07	19 (17.9)	63 (19.8)	0.05
Psychosis	20 (18.9)	1200 (25.2)	0.15	20 (18.9)	66 (20.8)	0.05
Bipolar disorder	32 (30.2)	1677 (35.2)	0.11	32 (30.2)	99 (31.1)	0.02
Tobacco use disorder	56 (52.8)	2887 (60.6)	0.16	56 (52.8)	177 (55.7)	0.06
Opioid use disorder	26 (24.5)	1173 (24.6)	<0.01	26 (24.5)	86 (27.0)	0.06
Liver disease	24 (22.6)	1972 (41.4)	0.41	24 (22.6)	65 (20.4)	0.05
Chronic kidney disease	11 (10.4)	1190 (25.0)	0.39	11 (10.4)	32 (10.1)	0.01

© 2024 Bernstein EY et al. *JAMA Netw Open.*

Prior alcohol withdrawal	41 (38.7)	2018 (42.4)	0.08	41 (38.7)	114 (35.8)	0.06
Alcohol-related comorbidities	12 (11.3)	1424 (29.9)	0.47	12 (11.3)	35 (11.0)	0.01
Remote MAUD use	*	*	0.24	*	*	0.03
Past-year primary care visit	62 (58.5)	2707 (56.8)	0.03	62 (58.5)	192 (60.4)	0.04
Past-year mental health visit	16 (15.1)	667 (14.0)	0.03	16 (15.1)	51 (16.0)	0.03
Past-year hospitalizations						
None	41 (38.7)	1139 (23.9)		41 (38.7)	112 (35.2)	
One	19 (17.9)	769 (16.1)	0.36	19 (17.9)	55 (17.3)	0.08
Greater than 1	46 (43.4)	2855 (59.9)		46 (43.4)	151 (47.5)	
Past-year ED visits						
None	20 (18.9)	1050 (22.0)		20 (18.9)	55 (17.3)	
One	21 (19.8)	828 (17.4)	0.09	21 (19.8)	75 (23.6)	0.09
Greater than 1	65 (61.3)	2885 (60.6)		65 (61.3)	188 (59.1)	
Past-year behavioral therapy	33 (31.1)	1215 (25.5)	0.13	33 (31.1)	107 (33.6)	0.05
Past-30-day behavioral therapy	*	*	0.03	*	*	0.09
**Hospitalization factors**						
Elixhauser readmission index, mean (SD)	17.6 (14.6)	22.7 (14.5)	0.35	17.6 (14.6)	18.5 (12.7)	0.07
Inpatient addiction medicine or psychiatry, n (%)						
None	40 (37.7)	3433 (72.1)		40 (37.7)	113 (35.5)	
Psychiatric hospital	23 (21.7)	324 (6.8)	0.75	23 (21.7)	78 (24.5)	0.04
Psychiatry/addiction medicine at general hospital	43 (40.6)	1006 (21.1)		43 (40.6)	127 (39.9)	
Self-directed discharge, n (%)	*	*	0.36	*	*	0.03
Length of stay, mean (SD)	7.1 (5.3)	4.8 (4.5)	0.46	7.1 (5.3)	6.8 (6.1)	0.04
Discharge month	*	*	0.39	*	*	0.16

MAUD = Medications for alcohol use disorder (naltrexone, acamprosate, disulfiram), SMD = Standardized mean difference, SD = Standard deviation, ED = Emergency department
*Indicates redacted due to CMS cell suppression policy threshold for display of data (values <11)
[A] Self-reported and imputed race and ethnicity were determined using the Research Triangle Institute race code. Other includes Asian, North American Native, and unknown, grouped due to small sample size.

© 2024 Bernstein EY et al. *JAMA Netw Open.*

Supplemental Table 8. Characteristics before and after propensity matching in sensitivity analysis restricted to patients with more than one discharge medication fill

	Before matching			After matching		
Characteristic	Discharge MAUD (N=192)	No discharge MAUD (N=3,829)	SMD	Discharge MAUD (N=192)	No discharge MAUD (N=576)	SMD
**Demographics**						
Age, mean (SD), y	53.5 (12.5)	54.2 (13.3)	0.05	53.5 (12.5)	53.5 (13.3)	<0.01
Sex, n (%)						
Female	79 (41.1)	1395 (36.4)	0.10	79 (41.1)	227 (39.4)	0.04
Male	113 (58.9)	2434 (63.6)		113 (58.9)	349 (60.6)	
Race/ethnicity, n (%)[A]						
Black	*	*	0.16	*	*	0.10
Hispanic	*	*		*	*	
White	*	*		*	*	
Other	*	*		*	*	
Social Deprivation Index, mean (SD)	47.2 (26.1)	49.0 (28.1)	0.07	47.2 (26.1)	47.1 (27.7)	<0.01
Region, n (%)						
Northeast	59 (30.7)	1045 (27.3)	0.11	59 (30.7)	187 (32.5)	0.05
Midwest	42 (21.9)	946 (24.7)		42 (21.9)	130 (22.6)	
South	69 (35.9)	1309 (34.2)		69 (35.9)	196 (34.0)	
West	22 (11.5)	529 (13.8)		22 (11.5)	63 (10.9)	
Medicaid dual-eligibility, n (%)	135 (70.3)	2799 (73.1)	0.06	135 (70.3)	394 (68.4)	0.04
Reason for entitlement, n (%)						
Age	31 (16.1)	644 (16.8)	0.02	31 (16.1)	87 (15.1)	0.03
Disability	161 (83.9)	3185 (83.2)		161 (83.9)	489 (84.9)	
**Clinical factors,** n (%)						
Anxiety disorder	117 (60.9)	2271 (59.3)	0.03	117 (60.9)	351 (60.9)	<0.01
Depression	180 (93.8)	3296 (86.1)	0.26	180 (93.8)	545 (94.6)	0.04
Posttraumatic stress disorder	36 (18.8)	613 (16.0)	0.07	36 (18.8)	118 (20.5)	0.04
Psychosis	49 (25.5)	1309 (34.2)	0.19	49 (25.5)	137 (23.8)	0.04
Bipolar disorder	79 (41.1)	1669 (43.6)	0.05	79 (41.1)	250 (43.4)	0.05
Tobacco use disorder	98 (51.0)	2280 (59.5)	0.17	98 (51.0)	298 (51.7)	0.01
Opioid use disorder	44 (22.9)	882 (23.0)	<0.01	44 (22.9)	148 (25.7)	0.06
Liver disease	38 (19.8)	848 (22.1)	0.06	38 (19.8)	113 (19.6)	0.00
Chronic kidney disease	19 (9.9)	695 (18.2)	0.24	19 (9.9)	55 (9.5)	0.01

© 2024 Bernstein EY et al. *JAMA Netw Open.*

Prior alcohol withdrawal	64 (33.3)	730 (19.1)	0.33	64 (33.3)	189 (32.8)	0.01
Alcohol-related comorbidities	20 (10.4)	438 (11.4)	0.03	20 (10.4)	58 (10.1)	0.01
Remote MAUD use	*	*	0.26	*	*	0.04
Past-year primary care visit	116 (60.4)	2225 (58.1)	0.05	116 (60.4)	351 (60.9)	0.01
Past-year mental health visit	42 (21.9)	813 (21.2)	0.02	42 (21.9)	120 (20.8)	0.03
Past-year hospitalizations						
None	63 (32.8)	1249 (32.6)		63 (32.8)	187 (32.5)	
One	43 (22.4)	737 (19.2)	0.08	43 (22.4)	139 (24.1)	0.04
Greater than 1	86 (44.8)	1843 (48.1)		86 (44.8)	250 (43.4)	
Past-year ED visits						
None	38 (19.8)	901 (23.5)		38 (19.8)	103 (17.9)	
One	36 (18.8)	762 (19.9)	0.11	36 (18.8)	107 (18.6)	0.05
Greater than 1	118 (61.5)	2166 (56.6)		118 (61.5)	366 (63.5)	
Past-year behavioral therapy	66 (34.4)	1148 (30.0)	0.09	66 (34.4)	184 (31.9)	0.05
Past-30-day behavioral therapy	19 (9.9)	414 (10.8)	0.03	19 (9.9)	66 (11.5)	0.05
**Hospitalization factors**						
Discharge diagnosis category, n (%)						
Alcohol	106 (55.2)	1610 (42.0)		106 (55.2)	307 (53.3)	
Mood disorder	64 (33.3)	1188 (31.0)	0.41	64 (33.3)	210 (36.5)	0.07
Other mental health	22 (11.5)	1031 (26.9)		22 (11.5)	59 (10.2)	
Elixhauser readmission index, mean (SD)	17.8 (12.5)	22.5 (13.2)	0.37	17.8 (12.5)	18.1 (11.5)	0.03
Inpatient addiction medicine or psychiatry, n (%)						
None	42 (21.9)	1326 (34.6)		42 (21.9)	130 (22.6)	
Psychiatric hospital	85 (44.3)	1473 (38.5)	0.29	85 (44.3)	272 (47.2)	0.08
Psychiatry/addiction medicine at general hospital	65 (33.9)	1030 (26.9)		65 (33.9)	174 (30.2)	
Self-directed discharge, n (%)	*	*	0.13	*	*	0.03
Length of stay, mean (SD)	8.2 (6.8)	7.0 (7.0)	-0.17	8.2 (6.8)	7.9 (7.7)	-0.03
Discharge month	*	*	0.27	*	*	0.14

MAUD = Medications for alcohol use disorder (naltrexone, acamprosate, disulfiram), SMD = Standardized mean difference, SD = Standard deviation, ED = Emergency department
*Indicates redacted due to CMS cell suppression policy threshold for display of data (values <11)
[A] Self-reported and imputed race and ethnicity were determined using the Research Triangle Institute race code. Other includes Asian, North American Native, and unknown, grouped due to small sample size.

© 2024 Bernstein EY et al. *JAMA Netw Open.*

Supplemental Figure 1. Propensity score distribution before and after 3:1 matching in primary analysis

Panel A. Propensity score distribution prior to matching in full cohort (n=9,834)

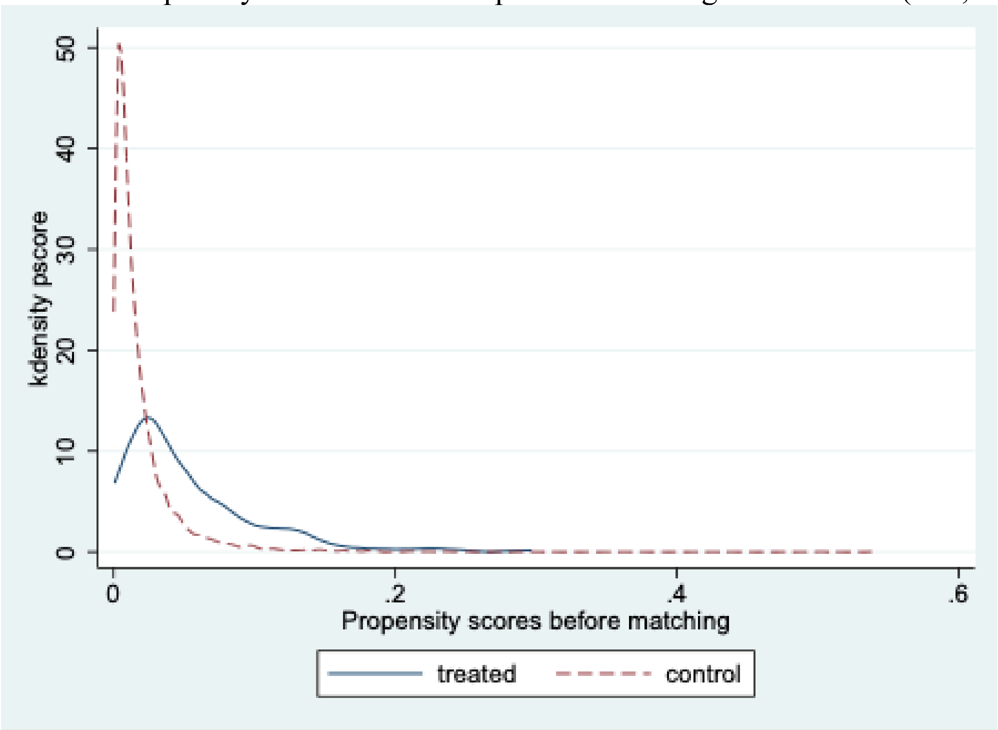

Panel B. Propensity score distribution after matching (n=768)

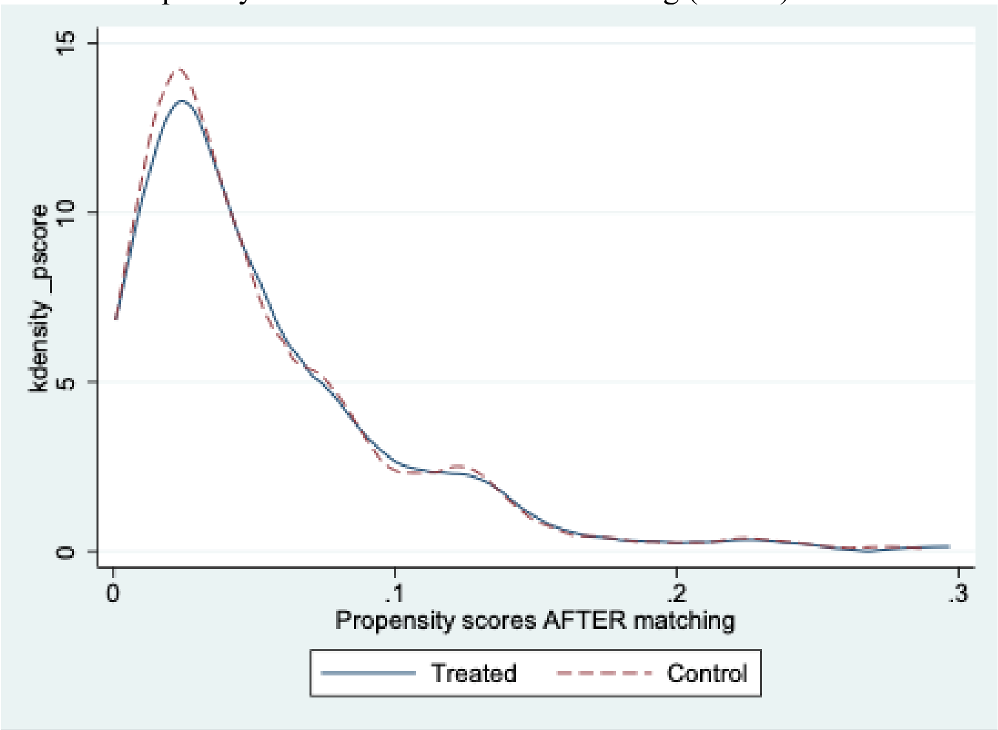

© 2024 Bernstein EY et al. *JAMA Netw Open.*

Supplemental Figure 2. Propensity score distribution before and after 3:1 matching in sensitivity analysis restricted to a single hospitalization per patient

Panel A. Propensity score distribution prior to matching (n=6,794)

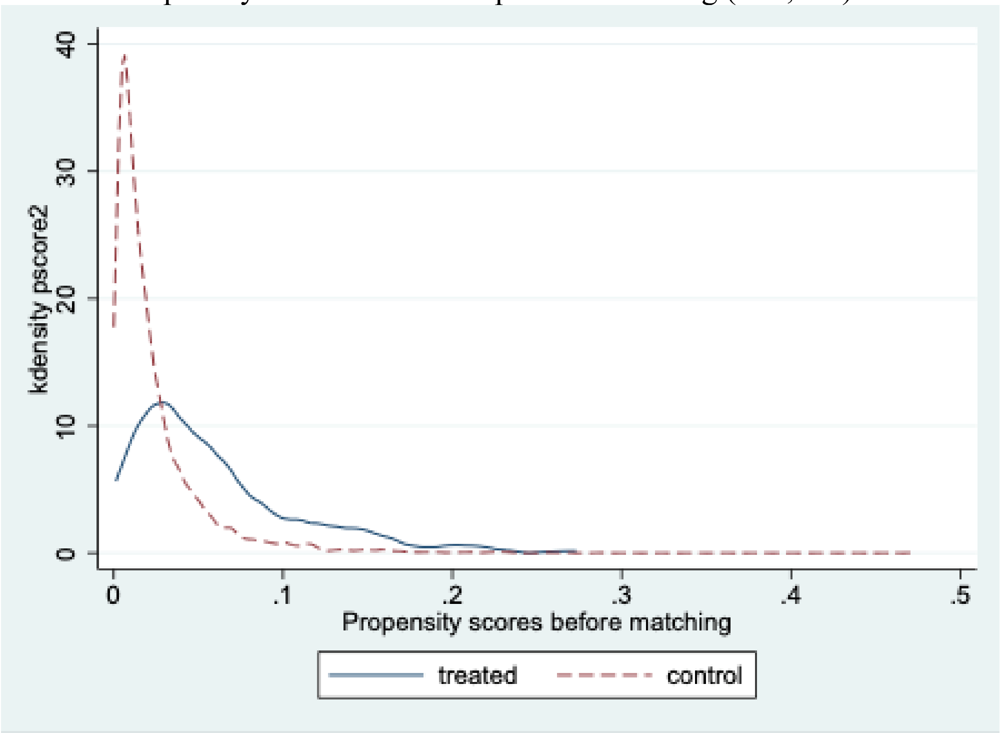

Panel B. Propensity score distribution after matching (n=664)

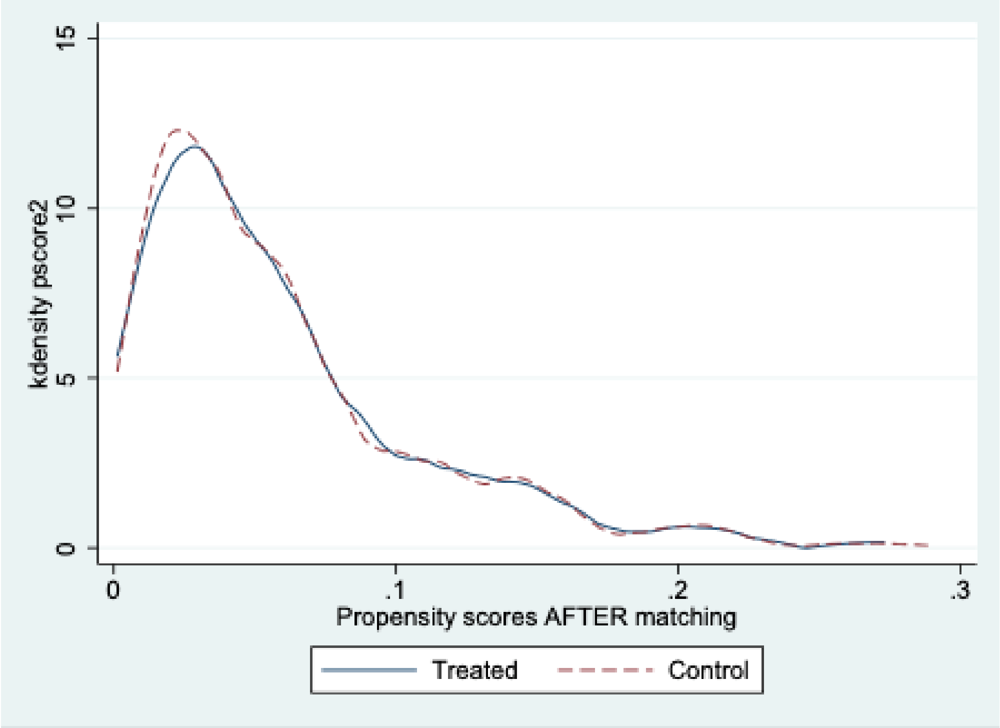

© 2024 Bernstein EY et al. *JAMA Netw Open.*

Supplemental Figure 3. Propensity score distribution before and after 3:1 matching in sensitivity analysis restricted to primary AUD discharge diagnoses

Panel A. Propensity score distribution prior to matching (n=4,869)

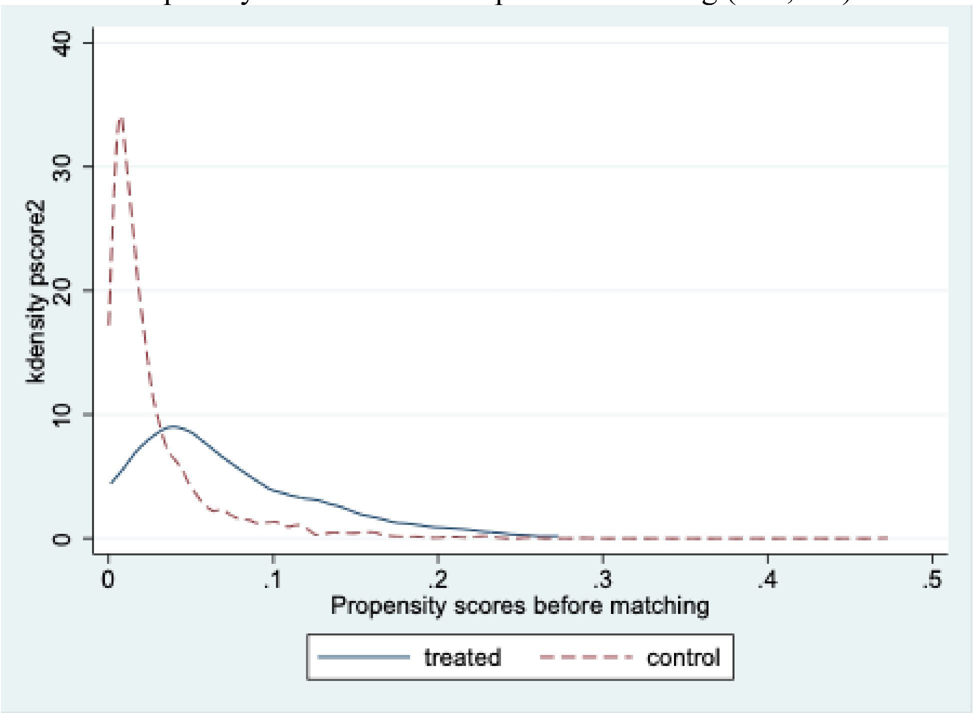

Panel B. Propensity score distribution after matching (n=304)

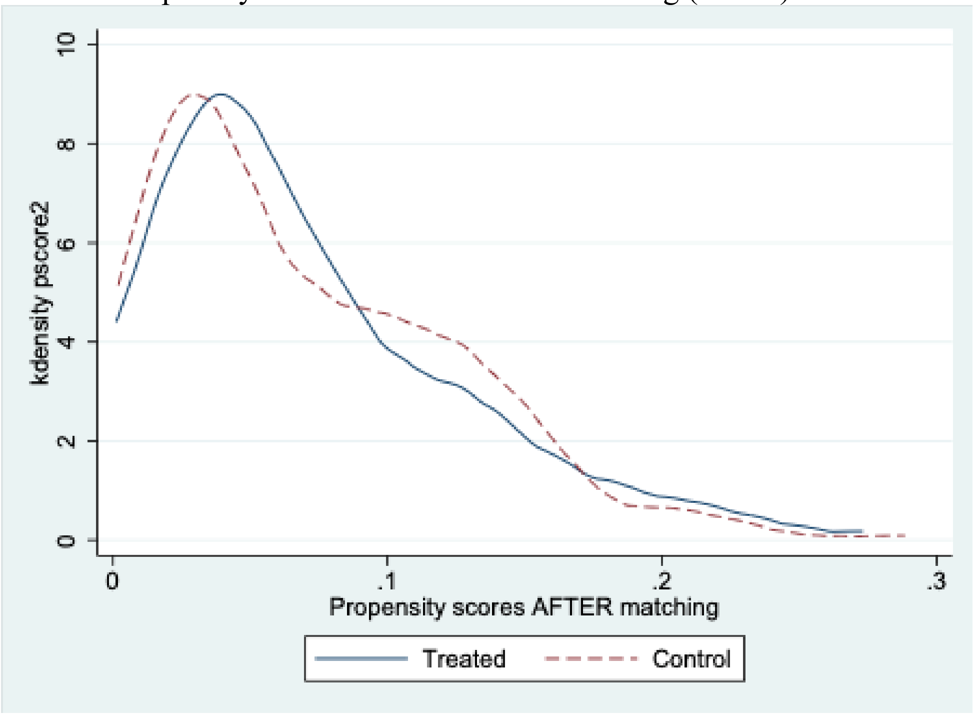

© 2024 Bernstein EY et al. *JAMA Netw Open.*

Supplemental Figure 4. Propensity score distribution before and after 3:1 matching in sensitivity analysis restricted to patients with more than one discharge medication fill

Panel A. Propensity score distribution prior to matching (n=4,021)

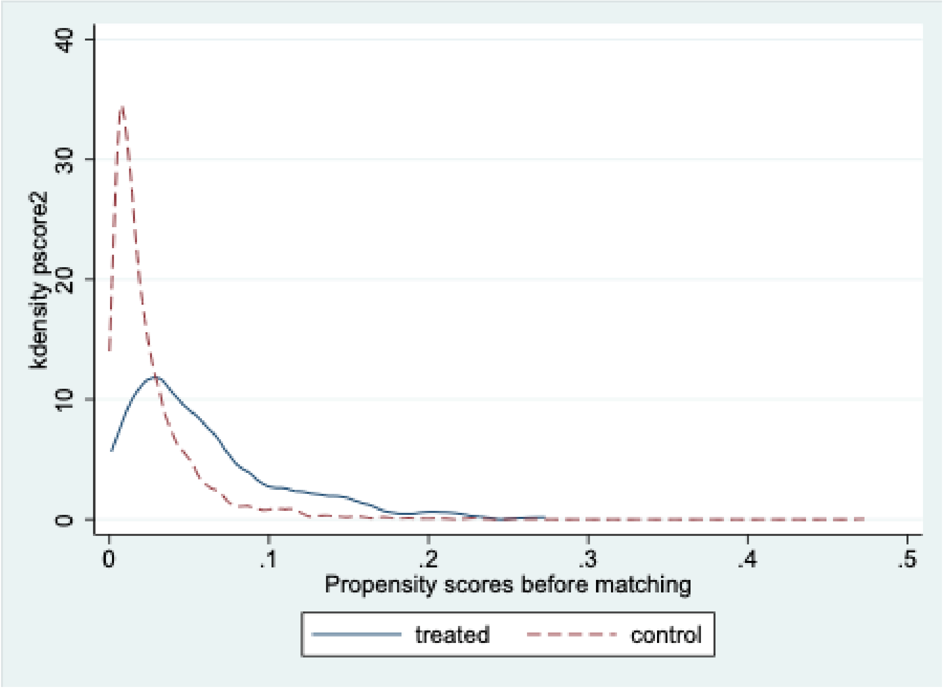

Panel B. Propensity score distribution after matching (n=768)

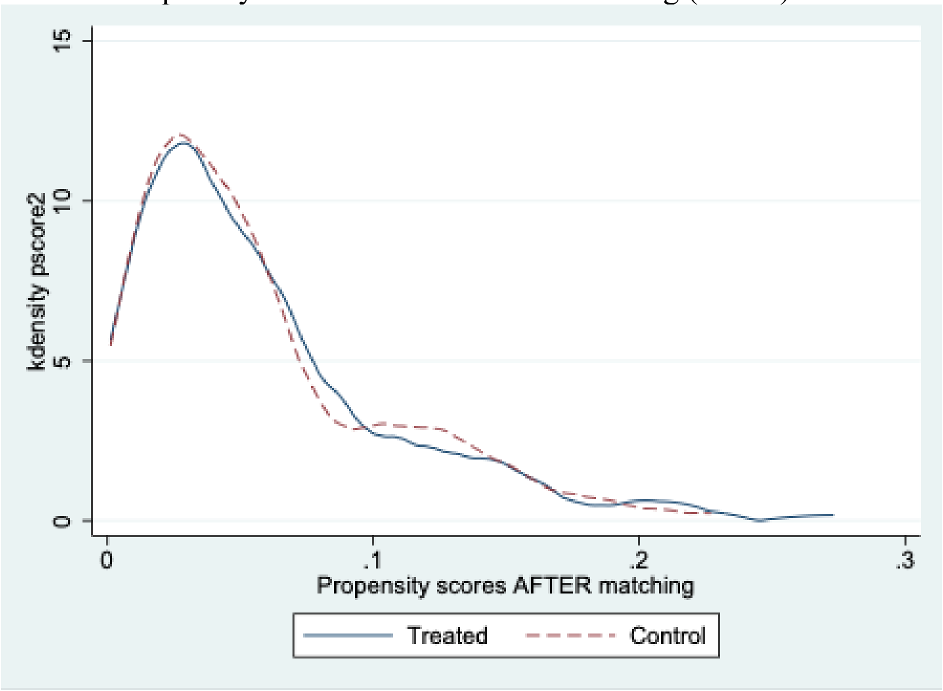

© 2024 Bernstein EY et al. *JAMA Netw Open.*
