# Supplementary material for: Outcomes After Initiation of Medications for Alcohol Use Disorder at Hospital Discharge
Source: JAMA Netw Open. 2024 Mar 29;7(3):e243387. doi: 10.1001/jamanetworkopen.2024.3387 (PMC10980961; doi:10.1001/jamanetworkopen.2024.3387)
Supplement: Supplement 2. — Data Sharing Statement [file jamanetwopen-e243387-s002.pdf]

Data Sharing Statement

Bernstein. Outcomes after initiation of medications for alcohol use disorder at hospital discharge. *JAMA Netw Open*. Published online March 29, 2024. doi:10.1001/jamanetworkopen.2024.3387

Data
Data available: No

Additional Information
Explanation for why data not available: Prohibited by CMS data use agreement.
